# ‘More constricting than inspiring’ — GPs find chronic care programmes of limited clinical utility. A qualitative study

**DOI:** 10.3399/bjgpopen18X101591

**Published:** 2018-05-30

**Authors:** Mads Aage Toft Kristensen, Tina Drud Due, Bibi Hølge-Hazelton, Ann Dorrit Guassora, Frans Boch Waldorff

**Affiliations:** 1 PhD Student, Department of Public Health, Research Unit for General Practice and Section for General Medicine, Copenhagen, Denmark; 2 GP, Southern Køge Medical Centre, Region Zealand, Denmark; 3 Postdoctoral Researcher, Department of Public Health, Research Unit for General Practice and Section for General Medicine, Copenhagen, Denmark; 4 Professor, Zealand University Hospital, Region Zealand, Denmark; 5 Department of Regional Health Research, University of Southern Denmark, Odense, Denmark; 6 Associate Professor, Department of Public Health, Research Unit for General Practice and Section for General Medicine, Copenhagen, Denmark; 7 Professor, Research Unit of General Practice, Institute of Public Health, University of Southern Denmark, Odense, Denmark; 8 Associate Professor, Department of Public Health, Research Unit for General Practice and Section for General Medicine, Copenhagen, Denmark

**Keywords:** Multimorbidity, chronic disease, general practice, self-care, continuity of patient care, disease management

## Abstract

**Background:**

As in other countries, Danish health authorities have introduced disease management programmes (DMPs) to improve care quality. These contain clinical practice guidelines (CPGs) and guidelines for patient stratification based on doctors’ assessments of disease severity and self-care. However, these programmes are challenged when patients have complex chronic conditions.

**Aim:**

To explore how GPs experience the clinical applicability of disease management programmes for patients with multiple chronic conditions and lowered self-care ability.

**Design & setting:**

A qualitative study from general practice, conducted in rural areas of Denmark with economically disadvantaged populations.

**Method:**

Data were collected through case-based, semi-structured interviews with 12 GPs. The principles of systematic text condensation were used in the analysis.

**Results:**

GPs found DMPs inadequate, particularly for patients with multiple conditions and lowered self-care ability. Their experience was that adhering to multiple programmes’ CPGs resulted in too much medication, conflicting treatments, an overload of appointments, and fragmented health care. They disregarded stratifying according to guidelines because they deemed stratification criteria to reflect neither patients’ need for self-care support, nor flexible referral options to hospitals and municipalities. Therefore, GPs were often solely responsible for treatment of patients with very complex chronic conditions.

**Conclusion:**

GPs found DMPs to be of limited clinical applicability due to challenges related to CPGs, patient stratification, and lack of adequate health services to support patients with complex healthcare needs. To increase the benefits of these programmes, they should be more flexible, and adjusted to the needs of patients with multiple chronic conditions and lowered self-care ability.

## How this fits in

DMPs are implemented internationally to optimise the management of chronic conditions, but little is known about GPs’ clinical experiences using them. This qualitative study found that Danish GPs viewed current DMPs as of limited clinical applicability when caring for patients with multiple chronic conditions or lowered self-care ability. This was due to challenges related to CPGs, patient stratification, and lack of adequate health services. DMPs should be altered to include patients with complex health problems, or the use of procedural standards like the DMPs should be discarded altogether for this high complexity patient group.

## Introduction

Worldwide, health authorities have standardised chronic care around DMPs, to ensure better quality of disease management and appropriate utilisation of healthcare resources.^1^ Since 2008, and inspired by the Chronic Care Model,^2^ the Danish health system has introduced DMPs for chronic conditions^3^ such as type 2 diabetes (T2DM), heart failure, and dementia. Drawn up by health professionals and local authorities, the DMPs encompass available CPGs (describing evidence-based care, including systematised diagnostics, treatment, follow-up, and support for self-care), and describe the interdisciplinary and cross-sectoral organisation between general practice (Box 1), municipalities (local authorities in the UK), and hospitals.^4^ By means of stratification, the DMPs divide patients into groups according to disease severity and care needs. This should ensure standardised adjustment of chronic care to the needs of different patients throughout the health system,^3^ by taking account of disease intensity and complexity, comorbidity, and patients’ self-care ability, which includes their capacity to follow treatment. For example, patients with T2DM should usually be managed in general practice, but also consult specialist care in case of increasing disease severity, and municipal self-care support in case of poor self-care ability (Table 1).

**Box 1.** General practice in Denmark.

Almost the entire Danish population is registered with a GP for primary health care, which is tax-financed and free at the point of use. GPs are private entrepreneurs regulated through collective agreements between the Danish regions and the organisation of GPs.^5^ Patients need referrals from their GPs to consult hospital specialists and to access municipal educational self-care support. Consequently, GPs in Denmark act as gatekeepers to other health services and play a key role in chronic care, which is organised through disease management programmes (DMPs).

**Table 1.** An example of how GPs are expected to stratify patients with type 2 diabetes, determining the level of chronic care^6^

		Disease regulation	
		Well	Poor
Self-care	High	General practice	General practice Specialist care
	Low	General practice Self-care support	General practice Specialist care Self-care support

However, Danish studies have found that some patients with less severe T2DM receive specialist care, while others with severe T2DM remain in general practice.^7,8^ In addition, an increasing number of patients have multiple concurrent chronic diseases^9^ and may be covered by several DMPs simultaneously. Adding to the complexity is that assessments of patients’ self-care ability are not well-established, since the concept of self-care is contested and has multiple definitions.^10^ In most cases, GPs are responsible for stratification, self-care assessments, and implementing relevant DMPs for individual patients. Therefore, it is important to explore GPs’ clinical experiences with DMPs, especially in the care of patients with multiple chronic conditions and lowered self-care ability. The literature on this subject is sparse.

### Aim

This article explores how GPs experience the clinical applicability of DMPs in the management of patients with multiple chronic conditions and lowered self-care ability.

## Method

GPs were recruited from two rural municipalities in south-eastern Denmark that are characterised by a population with lower socioeconomic status and a high prevalence of chronic conditions. The sampling was a 2-step process. First, one of the researchers, who is also a GP, gave a presentation at three local GP meetings, which resulted in four GPs agreeing to participate. Second, with the intention to include 12 GPs in total, an additional 10 GPs were consecutively invited among all 55 GPs in the study area. To provide maximal variation, the GPs were purposively sampled based on age, sex, practice size, and location (Table 2). Of these, eight agreed to participate.

**Table 2.** Personal and demographic details of the GPs who participated in the study, *n* = 12

**Median age, years (range)**	56 (37–69)
**Sex, *n***	
Male	6
Female	6
**Time in practice, years (range)**	16 (1–41)
**Practice size**	
1 GP	6
2 GPs	6
**Practice location**	
Village, <5000 inhabitants	3
Town, ≥﻿5000 inhabitants	9
**Distance from practice to hospital**	
≤﻿30 minutes' drive, *n* (range)	5 (2–27)
>30 minutes' drive, *n* (range)	7 (35–51)

In 2015, the researcher conducted and audio-recorded individual, semi-structured interviews with participating GPs at their practices. The interviews began with questions linked to three anonymised case patients, identified by each GP in advance, and were followed up with questions on broader experiences.

All patient cases satisfied the following selection criteria:

- diagnosis of T2DM;
- diagnosis of ≥1 additional chronic condition; and
- the GP experienced the patient having difficulty following treatment, as a proxy of lowered self-care.

T2DM was chosen as a criterion because of its high prevalence, and because it was the first disease to have a DMP. In addition, the patient cases had prevalent chronic diseases often combined with mental disorders, addiction problems (Table 3), or complicating social circumstances. Moreover, age and sex of the selected patients were well distributed, so the study data concern a broad spectrum of patients. Boxes 2 and 3 illustrate typical scenarios where GPs found these patients were facing challenges following recommended treatment.

**Table 3.** Profile of the patient cases that informed discussion in the GP interviews, *n* = 36

**Age, years**		
Mean	62.5	
Range	37–81	
**Sex, *n* (%)**		
Male	21	(58)
Female	15	(42)
**Chronic conditions, *n* (%)**		
Diabetes	36	(100)
Heart disease	18	(50)
Mental disorder	16	(44)
Obesity	14	(39)
Addiction (alcohol or cannabis)	9	(25)
Musculoskeletal disorders	8	(22)
Respiratory disease	4	(11)

**Box 2.** Example of a patient with concurrent mental and somatic diseases. (GP 7)

Peter is a middle-aged man with schizophrenia and periodic alcohol misuse, who is overweight. He also suffers from type 2 diabetes, heart failure, and chronic obstructive pulmonary disease. His GP had tried to refer Peter to hospital several times, but Peter often cancels or leaves the hospital because he cannot cope in the large hospital setting. The GP finds that Peter has an unbearable feeling of insecurity which is related to his psychiatric disorder. Therefore, the GP manages Peter’s chronic conditions, although she does not see this as the best solution for Peter. They are in weekly contact and Peter gets appointments at very short notice, because he has so many diseases to deal with and his conditions easily exacerbate.

**Box 3.** Example of a patient with concurrent somatic diseases. (GP 6)

John is a retired manual worker in his early seventies who has diabetes and possibly dementia, but he refuses further medical examination. He often shows up at the GP’s surgery without an appointment. The GP has talked frequently to John and his wife about improving disease regulation through diet and exercise, but John has not managed to change his habits. Recently, John’s wife has been diagnosed with cancer and cannot support John as much as before. John lives in the countryside and he disagrees with his wife’s suggestion of moving to the nearby town, although he is at risk of losing his driver’s licence.

The interviews were transcribed verbatim. Systematic text condensation was used in the analysis of data, which is a pragmatic phenomenological approach that aims to derive knowledge from everyday experience, and is suited to a cross-case analysis of a phenomenon to identify new descriptions and concepts.^11^

The analysis had four steps:

1. establishment of themes for coding;
2. classification of the meaning units;
3. abstraction and condensation; and
4. synthesis into consistent descriptions and concepts.

The research team comprised three medical doctors (of which two were GPs), a nurse, and a Master of Science in Public Health, all of whom identified the themes. The analysis was inductive, and the researcher performed open coding of all interviews by hand. The process was mentored by two other researchers, who also independently coded 10 and five interviews respectively. Through comparison of codes, the researchers discussed the coding framework and interpretations until consensus was reached. This process also led to re-reading of data extracts and whole interviews. One of the two mentoring researchers participated in and supervised the condensation and synthesis. Other themes related to self-care are published elsewhere.^12^

## Results

All participating GPs emphasised that chronic care was a substantial part of their work. They had organised chronic care in general accordance with CPGs: they planned to see their patients annually for systematised, disease-specific consultations, and often delegated in-between check-ups to practice nurses. Nonetheless, this structure was difficult to maintain when patients had multiple chronic conditions or lowered self-care ability.

### GPs’ clinical experiences with CPGs in the DMPs

GPs found CPGs difficult to adhere to when patients had multiple chronic conditions or lowered self-care ability, due to four main GP-perceived patient challenges: too much medication; conflicting treatments; overload of healthcare appointments; and non-attendance. In cases of multiple chronic conditions, several DMPs were brought into play, and the recommended medication sometimes overwhelmed patients. Without any guidance from the DMPs, GPs often had to help patients prioritise between several medications to ensure adherence, even if this sometimes went against recommendations:

*If you follow guidelines, they must take more than 20 drugs, and the role of the GP is to reduce it to four or five drugs. Otherwise, the patients do not take it … which we observe from their electronic records. *(GP 9)

Further, CPG-recommended treatment of one disease could conflict with exacerbations of other diseases or psychosocial problems. One GP described how she felt compelled to treat a respiratory disease with oral glucocorticosteroids at the expense of worsening the patient’s diabetes:

*It is very difficult … She is so ill with her lungs that I occasionally must treat her with prednisolone … When she sits here and is hardly able to breathe, then her lungs come first.* (GP 7)

GPs also described that following the recommended treatment of concurrent DMPs added up to a high number of healthcare appointments, both within and outside of general practice. Many patients were not able or willing to attend so many appointments. When several DMPs were used for one patient, some GPs bundled check-ups, although this goes against CPGs and GP financial reimbursement agreements:

*I try to group the chronic diseases. If a patient has hypertension and diabetes, we cover all of it in one consultation … In this way, they* [patients with multimorbidity] *get two or three annual check-ups for the major things.* (GP 3)

Non-attendance at scheduled check-ups for chronic conditions or attendance without an appointment were common challenges with patients who had lowered self-care ability. Most GPs mentioned that they would usually squeeze these patients in between scheduled appointments if they had specific worries about their health and were aware of their challenges attending appointments. When the patient's major problem was insufficient self-care ability to adapt their lifestyle to the disease, the GPs found the CPGs of little use, because their focus on medication and regular examinations could not improve disease regulation. They found few, if any, options in the CPGs of self-care support for patients with complex health problems:

*They might reach the target for a period, where it’s only about putting the right pill in their mouth, but at some point they get difficult to treat, because they don’t do much else than taking a pill for their disease. *(GP 8)

### GPs’ clinical experiences with stratification criteria in the DMPs

The DMPs require GPs to stratify patients according to assessments of the patient’s self-care ability and disease severity based on biomedical parameters. Thus, stratification forms the cornerstone of planning chronic care and may result in referral to other health professionals (Table 1). While most of the GPs were familiar with the concept of stratification, they rarely stratified patients as described in the DMPs. The GPs found stratification neither feasible nor beneficial for many patients, especially those with multiple chronic conditions and lowered self-care ability. As with CPGs, the complexity of these patients called for individualised judgments about the need for care that had to be continuously reconsidered. The biomedical focus of the DMPs was not compatible with the GPs’ whole-person approach to patients, and some GPs saw stratification as an externally imposed interference in the patient–doctor relationship:

*I have never liked to pigeonhole patients. It’s completely unrealistic. If we have to treat them equally, we must give different treatments. Twenty per cent of patients might fit into some boxes, but the others don’t. To me, it* [stratification] *is more constricting than inspiring. *(GP 5)

Further, the GPs found stratification had very little clinical relevance for patients with complex needs, because most referral options were inadequate for these patients.

### Hospitals and DMPs

The GPs found that hospitals did not meet the needs of patients with multiple chronic conditions and lowered self-care ability, because they focused on specific diseases and objective measures rather than the patient’s overall situation. GPs also found that many hospital clinics were staffed with nurses without authority to deviate from CPGs or confer with specialists outside their field. As a result, patients would often end up attending several clinics in different hospitals, which risked appointment overload and non-attendance:

*The diabetes outpatient clinic does nothing but adjust the medications to improve the blood sugar values … They have no additional programmes to support the patients. *(GP 7)

Further, the GPs found that health professionals in hospitals did not adjust treatment to the individual needs of patients with few resources, nor did they focus on self-care support, which they considered often to be the core challenge or the primary reason for the abnormal objective measurements that led to a hospital referral in the first place. The GPs also had experience with outpatient clinics dismissing patients if they did not show up at the scheduled time, or dismissing them for non-compliance:

*If* [the patient's diseases become poorly regulated or they develop complications]* by not following the treatment … these patients with lowered self-care are dismissed from the hospitals, because the specialists find that they are non-compliant and then it’s a job for the GP. *(GP 12)

For some patients with lowered self-care, a long journey to hospital was a barrier. The GPs suggested that the hospitals could adapt to the need of patients with few resources by establishing outpatient clinics with multiple specialties closer to patients’ homes:

*I wish to have outpatient clinics with different medical specialties closer to my patients … Many of my patients would appreciate avoiding the long journey, and I believe that it would enable more patients to visit a specialist.* (GP 5)

Instead of using stratification criteria in decisions about patient care and referral, the GPs made the decisions together with the patient by taking the patient’s preferences and resources into consideration. The GPs combined this with their own assumptions about the overall benefit of referral from a whole-person perspective. In these decisions, GPs often used their intuition and knowledge from their long-term doctor–patient relationship, giving less regard to objective biomedical parameters. The GPs said that when the patient and/or the GP decided against referral, or when patients were prematurely dismissed from the hospital, they had to manage patients’ complex chronic conditions in general practice. The GPs agreed that from a biomedical perspective, and based on the recommendations of the DMPs, they were not fully qualified to manage patients in this way. From a whole-person perspective, GPs perceived general practice as the most obvious place of treatment for patients with lowered self-care ability, because self-care support demanded knowledge of the patient’s situation without the further disruption to regular treatment that attendance at several specialised outpatient clinics can bring. However, within the current system, GPs lacked the financial and human resources to fulfil this task properly:

*These patients are thrown backwards and forwards between medical specialties in distant hospitals, and it makes it all worse, because they lose contact with general practice … These persons, who live here, would be worse off than if they had a single place with stability.* (GP 9)

### Municipalities and DMPs

According to stratification criteria, GPs should refer patients to municipal (local authority) health care if, in their assessment, the patients need self-care support. The GPs noted that many patients with multiple chronic conditions and lowered self-care ability required support to manage their conditions and to make lifestyle changes. According to the GPs, this requirement was not met by the municipalities, since the only referral option for self-care support was group-based educational programmes, which ran over a few months with no long-term follow-up. There was no other outreach programme or long-term self-care support:

*I would like the municipal self-care support to last longer than just such a one-night stand, so to speak. They go through all of it in one short course, but there should also be something to maintain the achievements.* (GP 1)

Moreover, many patients with limited educational attainment, mental disorders, or concurrent or disabling diseases did not fit into the educational programmes. The GPs found that more individual and proactive self-care support was missing. For example, they mentioned support from a social worker to attend healthcare appointments with the patients, to help them remember the prescribed regimen, and to understand the disease and the purpose of treatment:

*We should have contact with the municipality … to have a social worker who visits the patient and tries to support him to follow his regimen. It would probably give him some extra years to live. Now he might die soon.* (GP 9)

## Discussion

### Summary

The GPs in this study had generally organised chronic care in line with CPGs embedded in the DMPs. However, they found DMPs had several limitations, in particular for patients with multiple chronic conditions or lowered self-care ability. Firstly, to follow DMPs to the letter for concurrent diseases could mean that patients ended up with too much medication, conflicting treatments, an overload of appointments, fragmented health care, and no direction for self-care support. Secondly, GPs found stratification of these patients neither feasible nor beneficial, because the stratification criteria were too biomedical and did not reflect patients’ needs. Further, regarding referral to hospitals or municipal health services, the GPs found the available options were inadequate for this patient group. As a result, GPs often found themselves solely responsible for the treatment of patients with very complex chronic conditions in direct opposition to the intention of the DMPs.

### Strengths and limitations

To reduce the risk of conceptual blindness inherent in peer interviewing, the research team included health professionals from outside general practice throughout the study.^13,14^ However, participating GPs knew the interviewer’s identity as both GP and researcher, and expressed that they could talk more openly to a peer about clinical dilemmas than to an interviewer with another professional background.

This study took place in two areas of rural Denmark characterised by lower socioeconomic conditions, which might imply limited transferability of the findings to urban or more prosperous settings. However, a study from the UK found that GPs practicing in a wide range of socioeconomic settings experienced similar challenges with multimorbidity,^15^ suggesting that the challenges experienced by GPs in the present study are more likely due to dealing with patients with complex needs, rather than working with patients in lower socioeconomic settings.

The choice to let GPs pre-select three anonymised case patients facilitated discussion of concrete and comprehensive examples of their experiences, rather than abstract and generalised attitudes. To reduce the risk of the data focusing on only a few very complex patients, GPs were also asked broader questions to elicit more general views and experiences with this patient group. Further, as many of these results are also reported in other studies,^16–18^ it indicates that these findings are transferable to other settings.

### Comparison with existing literature

Challenges in chronic care management has been well described for years,^2,17,19^ and DMPs were thought of as a new approach to overcome barriers and suboptimal treatment in this area.^19^ However, these findings show that GPs found DMPs to be inadequate in clinical practice, especially for patients with complex health problems. Similarly, a recent Dutch study showed that GPs found CPGs less useful for complex cases and not adjustable to the needs of the individual patient.^16^ In fact, CPGs have been found to add to the complexity of patient care.^17^ Likewise, the GPs in the present study found that CPGs and stratification criteria embedded in the DMPs risked increasing the burden of treatment,^20^ with too much medication, or too many and fragmented healthcare appointments in general practice and hospitals for these patients, who are already at risk of being overburdened. This study also identified how GPs found this induced a risk of patients not adhering or attending, which correlates to findings by May *et al*, where increased burden of treatment, caused by expectations to adhere to complex treatment and self-monitoring regimes, led to structurally induced non-adherence and underutilisation of healthcare services, because patients felt overwhelmed.^20^ Hence, such treatment burdens may, in fact, even contribute to accumulated patient complexity over time.^21^ The literature on burden of treatment also describes how poor treatment outcomes may lead doctors to intensify treatment or refer patients to specialists with less knowledge of the patient, who then only increase doses rather than address the underlying difficulties in following the already prescribed treatment.^21^ This correlates to this study's findings of GPs experiencing a discrepancy between patients’ needs, and a focus on biomedical values in both DMPs’ stratification criteria and hospital services, when they perceived the main reason for a poor health status was lowered self-care ability. Thus, DMPs may sometimes cause new barriers and still leave the old unsolved. In the UK, a CPG for patients with multiple chronic conditions was recently published,^22^ but its clinical applicability needs further exploration.

The experience of the GPs in this study — that DMPs are inappropriate standardisation, which forces patients into boxes based on biomedical measurements rather than individualised treatment decisions — has also been identified elsewhere. Studies of the Quality and Outcomes Framework implemented in the UK showed that British GPs experienced a conflict between the systematic focus on self-management and other areas of professional responsibility, such as prioritising the biomedical aspects of care at the expense of exploring the patient’s perspective.^18^ Further, patients found themselves leaving consultations with unmet biomedical, informational, and emotional needs due to the external demand of structured chronic care.^23^ Regarding stratification, the GPs in this study found little, if any, clinical relevance for this categorisation as specified in the DMPs, and they experienced inadequate referral options to other health services, leading to fragmentation of care in some cases. Instead, they evaluated patients from a different angle when considering referral to other health services. To the GPs, their own evaluation had a purposeful clinical scope, focused on maximising the benefit to the whole person by including patient preferences and resources; it was more intuitive, built on knowledge from the long-term doctor—patient relationship, and less dependent on objective measurements or the directions of the DMPs. This practice resembles clinicians' use of 'mindlines' rather than guidelines, as identified by Gabbay and May, which are internalised tacit guidelines based on experience, collective collegial reinforcement, and negotiations at the individual patient consultation.^24^ The GPs in this study used their prior knowledge of the patient’s individual situation to accommodate patchy or ad hoc attendance at clinics, and to determine an individual course of treatment which combined recommendations from diverse CPGs and considerations about referral. Therefore, relational continuity^25^ seems crucial in the care of these patients throughout the healthcare system.

DMPs are an example of the standardisation movement in health care; they are procedural standards, which specify how processes are to be performed.^26^ Often — due to reasons like lack of knowledge, or lack of compliance, resistance, or adaption — such standardised protocols are not implemented as intended.^27^ This is also the case for the DMPs, where the GPs did not perceive them to encompass the complexities of their patients and, hence, did not always adhere to them. As in the present study, other studies have found that clinicians bend CPGs to prioritise the perceived needs and capacities of the individual patient.^28^ This raises the question of whether DMPs as a standard are too rigid to work in the care of patients with multimorbidity or lowered self-care ability. Loose standards with greater adaptability may work better than rigidly defined standards.^27^ In chronic care, there may be a case for having a greater degree of flexibility compared to management of acute illness, because treatment plans are not directed towards the unambiguous goal of a ‘cured’ patient, but instead directed towards making the best of an ongoing and open-ended illness.^28^ This adaptability and flexibility is not evident in the DMPs, but it could be argued that a 'bending' of stratification and CPGs may be necessary to make the standards work,^29^ and that it also illustrates a requirement for a deeper understanding of how clinicians reach medical decisions,^27^ which is not reflected in the DMPs’ stratification criteria. At the same time, as described within the standardisation literature, balance is needed between flexibility and rigidity, since too much flexibility may tip a standard into uselessness.^27^ Therefore, the question is whether it is most appropriate to allow a greater degree of flexibility to the current standardised programmes — to alter DMPs to include patients with multimorbidity or lowered self-care ability — and to the actual clinical decisionmaking process, or to discard the application of procedural standards like the DMPs altogether for this patient group.

Lastly, despite the recommendations of DMPs, the GPs in this study commonly experienced that many of the most complex patients were solely cared for in general practice, and they expressed a need for support in this task from both hospitals and municipalities. In its original form, The Chronic Care Model included coordinated support by a care manager for the most complex patients,^2^ but this is not specified in the Danish DMPs. The GPs also found that these patients needed sustained and more proactive chronic care, with better access to hospital specialists and extra support from social workers. This point was exemplified by the GPs’ experience of non-attendance as a major challenge in the care of patients with complex needs, which could not be prevented without closer collaboration with municipal health and social care services.

### Implications for research and practice

GPs’ reluctance to use stratification is an important finding, since it is a cornerstone in DMPs internationally. When GPs in this study evaluated patients for referral, it was mainly built on knowledge from a lengthy doctor–patient relationship, and less on objective and standardised criteria. Further investigation of GPs’ experiences with stratification is needed to confirm this finding. Moreover, the authors suggest exploring GPs’ alternative patient evaluation, in order to redesign and thereby increase the clinical utility of DMPs’ stratification guidelines. The GPs in this study found that DMPs did not support them in managing patients with complex health problems and lowered self-care ability. Hence, guidance for such patients should be considered in a redesign of DMPs. Likewise, given the importance of referral in DMPs, optimal referral options are vital in a redesign. To meet patients’ individual needs and ensure viable referrals, GPs requested new referral options to hospital and municipal health services, such as outreach services or outpatient clinics closer to patients’ residence, with a broader clinical scope and a focus on lowered self-care ability. When referral of patients with complex chronic conditions is not possible, GPs expressed a great need for innovative ways to collaborate more closely with social workers and hospital specialists, which ought to be elaborated on in future DMPs.
